# Early Immune Alterations in Adult Patients with Trauma According to Injury Severity: Cell-Death Patterns and Inflammatory Mediator Profiles

**DOI:** 10.3390/jcm15114371

**Published:** 2026-06-05

**Authors:** Sung-Joon Park, Jung-Youn Kim, Sora Yun, Si-Hwa Kim, Kap Su Han, Jong-Hak Park, Young-Hoon Yoon

**Affiliations:** 1 Department of Emergency Medicine, Korea University College of Medicine, Seoul 02841, Republic of Korea; 2 Department of Emergency Medicine, Korea University Guro Hospital, Seoul 08308, Republic of Korea; 3 Department of Emergency Medicine, Korea University Anam Hospital, Seoul 02841, Republic of Korea; 4 Department of Emergency Medicine, Korea University Ansan Hospital, Ansan 15355, Republic of Korea

**Keywords:** trauma, injury severity score, CD66b-positive granulocytes, apoptosis, T lymphocytes, interleukin-1 receptor antagonist, flow cytometry

## Abstract

**Background/Objectives**: Trauma triggers complex early immune responses. However, the relationship among trauma severity, changes in immune cell survival, and circulating inflammatory mediators remains unclear. This study compared early cell viability and death patterns in CD66b^+^ granulocytes, total T lymphocytes, and CD4^+^ and CD8^+^ T-cell subsets as well as inflammatory mediator levels between patients with non-severe and severe trauma. **Methods**: This single-center prospective observational study included 67 adult patients with trauma who were classified into non-severe and severe trauma groups according to the Injury Severity Score (ISS < 15 vs. ISS ≥ 15). Blood samples were obtained within 1 h of arrival at the emergency department. Flow cytometry was used to assess the viability, early apoptosis, late apoptosis, and necrosis in the leukocyte subsets. Serum concentrations of intercellular adhesion molecule-1 (ICAM-1), macrophage migration inhibitory factor (MIF), CD40 ligand (CD40L), and interleukin-1 receptor antagonist (IL-1ra) were measured using enzyme-linked immunosorbent assays. **Results**: The severe trauma group had a significantly lower proportion of early apoptotic CD66b^+^ granulocytes than the non-severe trauma group (2.9% [1.4–6.7] vs. 6.3% [3.7–10.9], *p* = 0.001), whereas the live, late apoptotic, and necrotic CD66b^+^ granulocyte fractions did not differ significantly between the two groups. Most T-cell death parameters were similar between the groups, although an exploratory increase in necrotic CD4^+^ T lymphocyte abundance was observed in the severe trauma group. IL-1ra levels were significantly higher in the severe trauma group than in the non-severe trauma group and were associated with ISS in both mediator-only and adjusted sensitivity regression analyses. **Conclusions**: Severe trauma was associated with reduced early apoptosis in the CD66b^+^ granulocyte compartment and elevated IL-1ra levels shortly after injury compared with non-severe trauma. These findings suggest that early immune alterations after severe trauma may involve compartment-specific granulocyte death patterns and counter-regulatory inflammatory responses rather than generalized changes across leukocyte populations.

## 1. Introduction

Trauma is a significant global health burden and a leading cause of death and disability, particularly in younger populations [1,2]. Although early trauma care has improved, post-traumatic complications, such as infection and multiple organ dysfunction, substantially affect the subsequent clinical course [3,4]. Therefore, characterizing the early immune response after trauma is clinically relevant because early immune alterations may contribute to organ dysfunction, infection, and adverse outcomes [4,5,6].

After traumatic injury, the host immune system is rapidly activated. The early immune response involves both inflammatory activation and counter-regulatory anti-inflammatory responses [4,7,8]. Post-traumatic immune responses cannot be entirely explained by a simple sequential transition from hyperinflammation to immune suppression, but instead involve simultaneous and heterogeneous alterations across distinct immune compartments [4,5,6]. Therefore, the emergency department and pre-hospital phases may provide important windows for assessing early immune changes following major injury [4,9]. However, the relationship between trauma severity and early cell death patterns in selected leukocyte compartments remains unclear.

Granulocytes are primary effector cells in the innate immune response to tissue injury, and changes in their viability and death patterns may influence the early inflammatory response after trauma [7,10]. Severe injury is associated with early alterations in neutrophil-related innate immune responses, including changes in neutrophil phenotype, function, and apoptosis [11,12,13,14]. Although neutrophil phenotype and apoptosis alterations after trauma have been investigated, initial viability and cell death patterns within the broader granulocyte compartment in relation to trauma severity remain incompletely characterized [11,12,14].

Trauma also affects the adaptive immune system, resulting in changes in T-lymphocyte numbers, subset composition, and functional responses following severe injury [15,16]. These adaptive immune changes may be dynamic and subset-specific rather than uniformly expressed across all T-cell populations [15,16]. Therefore, evaluating both granulocyte and T-cell subsets may clarify whether early post-traumatic immune alterations differ between the innate and adaptive immune compartments.

Investigating circulating inflammatory mediators can provide information on systemic processes that are not captured by leukocyte cell-death measurements alone [8,17]. In this study, IL-1ra, ICAM-1, CD40L, and MIF were selected to represent four biologically relevant but limited domains of early post-traumatic immune activation: counter-regulatory anti-inflammatory signaling, leukocyte–endothelial interaction, platelet-related thromboinflammation, and injury-related inflammatory activation [8,17,18,19]. This exploratory study was focused on hyperacute leukocyte cell-death patterns and a limited set of prespecified circulating mediators; therefore, other commonly used trauma-related biomarkers, including IL-6, IL-10, TNF-α, lactate, CRP, and additional coagulation, endothelial, or tissue-injury markers, were not included.

Moreover, how trauma severity is related to hyperacute leukocyte cell-death patterns and selected circulating mediator profiles remains incompletely defined [4,5,8,17]. To address this gap, this study examined cell viability and cell-death patterns in predefined leukocyte compartments, including CD66b^+^ granulocytes, total T lymphocytes, and CD4^+^ and CD8^+^ T-cell subsets, according to trauma severity. The primary objective was to compare cell viability and cell-death patterns between patients with non-severe trauma (Injury Severity Score [ISS] < 15) and those with severe trauma (ISS ≥ 15). The secondary objectives were to compare selected circulating inflammatory mediator profiles between the two groups and to explore the association between these mediators and ISS.

## 2. Materials and Methods

### 2.1. Study Design and Setting

**Figure 1:**
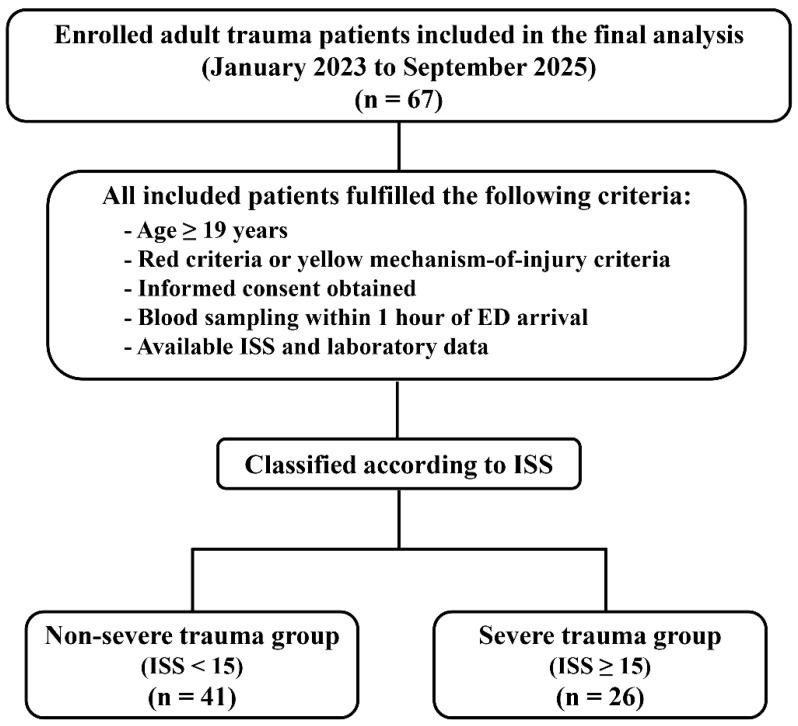
Flow diagram of the enrolled analytic cohort. This figure summarizes the derivation of the enrolled analytic cohort included in the present analysis. Because a complete prospective screening log was not maintained, this diagram does not represent the full screened trauma population during the study period. ED, emergency department; ISS, Injury Severity Score.

This was a single-center, prospective, observational study conducted in the emergency department of Korea University Guro Hospital, which is a regional emergency medical center serving the southwestern region of Seoul, with approximately 45,000 annual emergency department visits. It is one of the Seoul Metropolitan Government-designated definitive care centers providing definitive treatment for patients with severe trauma. Adult patients with trauma aged ≥ 19 years who visited the emergency department from January 2023 to September 2025 were considered eligible if they met any red criteria or any yellow criteria of the National Guideline for the Field Triage of Injured Patients [20]. The red criteria refer to patients at high risk of serious injury based on major injury patterns or abnormal mental status and vital signs, whereas the yellow mechanism-of-injury criteria refer to moderate-risk mechanisms such as high-risk motor vehicle crashes, separation from a transport vehicle with significant impact, pedestrian or bicycle rider injury with significant impact, or falls from a height of >10 feet. The emergency medical service judgment component of the yellow criteria was not used as an inclusion criterion. Patients were prospectively enrolled when they met these prespecified field-triage criteria, provided informed consent, and underwent research blood sampling within 1 h of emergency department arrival. Patients for whom informed consent and research blood sampling could not be completed within this 1 h window were not enrolled. All enrolled patients with available ISS and laboratory data were included in the final analytic cohort. A prospective screening log of all trauma patients, potentially eligible non-enrolled patients, reasons for non-enrollment, and patients with unavailable consent or samples was not maintained. Therefore, the flow diagram shown in Figure 1 summarizes the enrolled analytic cohort rather than the full screening denominator. Baseline demographic and clinical data, such as age, sex, injury severity, vital signs, and clinical outcomes, were collected for analysis. The manuscript was prepared in accordance with the Strengthening the Reporting of Observational Studies in Epidemiology (STROBE) statement, and the completed STROBE checklist is provided in Table S1.

### 2.2. Sample Collection and Processing

Whole blood samples were collected in ethylenediaminetetraacetic acid (EDTA)-containing tubes and serum separator tubes (SSTs) within 1 h of arrival at the emergency department. Samples collected in EDTA tubes were analyzed using flow cytometry. Samples collected in SST tubes were centrifuged to separate the serum, which was then aliquoted and stored at −80 °C until analysis.

### 2.3. Group Classification

Trauma severity was determined using the ISS. Patients with ISS ≥ 15 were classified into the severe trauma group, whereas those with ISS < 15 were classified into the non-severe trauma group, consistent with prior studies using ISS ≥ 15 to define major or severe trauma [21].

### 2.4. Laboratory Variables

The primary laboratory comparison focused on cell viability and Annexin V/propidium iodide-defined cell-death patterns in predefined leukocyte compartments, including CD66b^+^ granulocytes, total T lymphocytes, and CD4^+^ and CD8^+^ T-cell subsets, according to trauma severity. The levels of selected circulating inflammatory mediators, including ICAM-1, MIF, CD40L, and IL-1ra, were evaluated as secondary laboratory variables, and their association with ISS was explored. In this study, the CD66b^+^ granulocyte population was used as the target granulocyte compartment. The antibody panel was not designed to distinguish individual granulocyte subsets, neutrophil maturation states, or eosinophils. All laboratory variables were measured and analyzed by laboratory personnel who were blinded to the trauma severity group and clinical outcomes.

### 2.5. Flow Cytometric Staining and Acquisition

For surface marker staining, whole blood was incubated with fluorochrome-conjugated monoclonal antibodies against CD45, CD66b, CD3, CD4, and CD8 (BD Biosciences, San Jose, CA, USA) for 20 min at room temperature in the dark. Red blood cells were lysed using the BD Lysing Buffer (BD Biosciences, San Jose, CA, USA). Apoptotic cells were identified using an Annexin V Apoptosis Detection Kit (eBioscience, San Diego, CA, USA) according to the manufacturer’s instructions. Flow cytometric acquisition was performed on a BD LSRFortessa™ X-20 flow cytometer, and data were analyzed using the BD FACSDiva™ software (version 8.0.1; BD Biosciences, San Jose, CA, USA).

### 2.6. Flow Cytometry Gating Strategy and Apoptosis Analysis

Debris and non-cellular events were excluded by applying a polygon gate (P1) that excluded events with low forward scatter (FSC) and side scatter (SSC) signals, likely representing debris, while retaining the main leukocyte populations. This gating approach was applied consistently across all samples.

Subsequently, CD45^+^ leukocytes were identified from the P1 population (P2) based on CD45 expression and SSC distribution, whereas CD45-negative or dim non-leukocyte events were excluded. Within the CD45^+^ population, CD66b^+^CD3^−^ granulocytes were defined as P3, whereas CD66b^−^CD3^+^ T lymphocytes were defined as P4. T lymphocytes from the P4 gate were further subdivided into CD4^+^CD8^−^ and CD8^+^CD4^−^ T-cell subsets based on CD4 and CD8 expression.

Fluorescence minus one controls and single-stained controls were used during the initial optimization of the gating boundaries and compensation settings, respectively. Unstained controls were included in each experiment for the routine assessment of background fluorescence. A predefined gating template was consistently applied across the samples, with minimal adjustments made as needed according to the distribution of the control samples.

To minimize technical variability, sample processing, antibody staining, and data acquisition were performed according to the standardized protocols throughout the study. Instrument performance was evaluated according to the manufacturer’s quality control procedure before each acquisition day to minimize inter-day variability and potential instrument drift. The same acquisition template was used for all samples.

**Figure 2:**
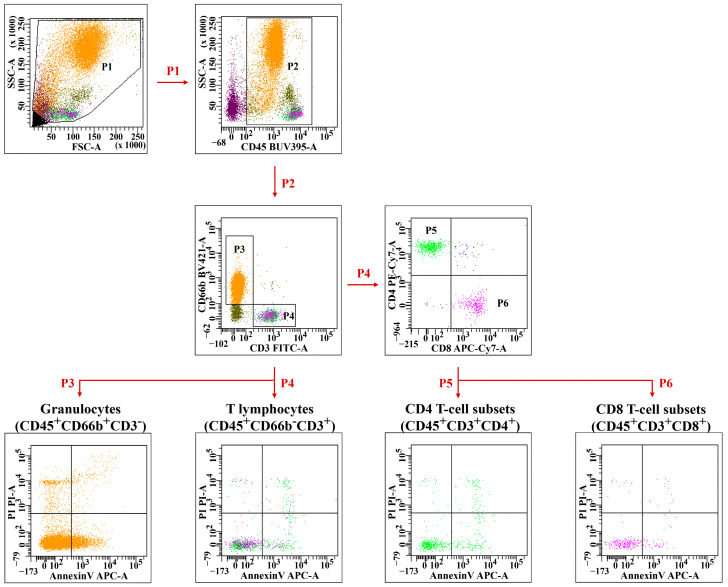
Flow cytometry gating hierarchy for CD66b^+^ granulocytes, total T lymphocytes, CD4^+^ and CD8^+^ T-cell subsets, and apoptosis analysis. First, debris and non-cellular events were excluded using FSC and SSC characteristics (P1). CD45^+^ leukocytes were then gated from the P1 population according to CD45 expression and SSC distribution (P2). Within CD45^+^ leukocytes, CD66b^+^CD3^−^ granulocytes were identified as P3, and CD66b^−^CD3^+^ T lymphocytes were identified as P4. T lymphocytes from the P4 gate were further subdivided into CD4^+^CD8^−^ T-cell subsets (P5) and CD8^+^CD4^−^ T-cell subsets (P6). Cell viability and cell-death states were assessed in each gated subset using Annexin V and propidium iodide staining. Colored dots indicate the corresponding gated cell populations and are used to visualize the gating hierarchy across panels. FSC, forward scatter; SSC, side scatter; CD, cluster of differentiation.

Cell viability and Annexin V/PI-defined cell-death states were assessed in each subset using Annexin V and propidium iodide (PI) staining according to established flow cytometric criteria [22,23]. Early apoptotic cells were defined by Annexin V^+^PI^−^, late apoptotic cells by Annexin V^+^PI^+^, and necrotic cells by Annexin V^−^PI^+^. Cell viability and death patterns were analyzed in CD45^+^CD66b^+^CD3^−^ granulocytes, CD45^+^CD66b^−^CD3^+^ T lymphocytes, and CD4^+^ and CD8^+^ T-cell subsets derived from the CD45^+^CD66b^−^CD3^+^ parent T-cell gate. The gating hierarchy and representative flow cytometry plots are shown in Figure 2.

### 2.7. Measurement of Circulating Inflammatory Mediators

Serum MIF, IL-1ra, CD40L, and ICAM-1 concentrations were measured using Quantikine enzyme-linked immunosorbent assay kits (R&D Systems, Minneapolis, MN, USA) according to the manufacturer’s instructions.

### 2.8. Study Size

Directly applicable preliminary data were not available to estimate the expected effect sizes for these hyperacute laboratory variables within the 1 h emergency department sampling window; therefore, a formal a priori sample size calculation was not performed. The study size was thus determined by feasibility during the study period, and the final analytic cohort included 67 adult patients with trauma who had available ISS and laboratory data and provided informed consent.

### 2.9. Statistical Analysis

There were no missing data for the variables included in the final analyses; therefore, no imputation was performed. Categorical variables were summarized as numbers and percentages, which were compared using the chi-square test or Fisher’s exact test, as appropriate. Continuous variables are expressed as means ± standard deviations for normally distributed data and as medians with interquartile ranges for non-normally distributed data. Normality was assessed using the Shapiro–Wilk test. Between-group comparisons were performed using the independent *t*-test for normally distributed continuous variables and the Mann–Whitney U test for non-normally distributed continuous variables. For laboratory between-group comparisons, *p*-values are presented without formal adjustment for multiple comparisons. No subgroup or interaction analyses were performed. The inflammatory mediator concentrations showed a skewed distribution; therefore, a natural log transformation was applied before analysis. Multiple linear regression analysis was performed with ISS as the dependent variable and log-transformed inflammatory mediator levels as the independent variables. For sensitivity analysis, an adjusted model was fitted by including age, sex, injury-to-sampling time, Glasgow Coma Scale (GCS), and systolic blood pressure. Regression results are presented as regression coefficients (β) with 95% confidence intervals. All statistical analyses were performed using IBM SPSS Statistics for Windows (version 29.0; IBM Corp., Armonk, NY, USA) and R software (version 4.5.3; R Foundation for Statistical Computing, Vienna, Austria). Two-sided *p* < 0.05 was considered statistically significant.

## 3. Results

### 3.1. Baseline Clinical Characteristics According to Trauma Severity

A total of 67 patients with trauma were included and classified into the non-severe trauma group (ISS < 15, *n* = 41) and the severe trauma group (ISS ≥ 15, *n* = 26) (Figure 1). The baseline characteristics of the patients are summarized in Table 1. Age and sex distributions were similar between the two groups (age, 54.8 ± 18.2 vs. 54.8 ± 19.8 years, *p* = 0.998; male sex, 73.2% vs. 73.1%, *p* = 1.000). The duration from emergency department arrival to blood sampling was not significantly different between the non-severe and severe trauma groups (45.0 ± 7.9 min vs. 47.5 ± 5.1 min, *p* = 0.144). In addition, the durations from injury occurrence to emergency department arrival and to blood sampling were not significantly different between the groups (41.0 [33.0–53.0] min vs. 41.5 [30.2–53.0] min, *p* = 0.541; 85.0 [76.0–105.0] min vs. 86.5 [75.5–105.2] min, *p* = 0.949, respectively). The severe trauma group had a lower Glasgow Coma Scale score and a higher Injury Severity Score than the non-severe trauma group. Respiratory rate was higher in the severe trauma group, and intensive care unit admission was more frequent in this group. No significant between-group differences were observed in the remaining baseline variables.

### 3.2. Cell Viability and Death Patterns of CD66b^+^ Granulocytes, Total T Lymphocytes, and CD4^+^ and CD8^+^ T-Cell Subsets Associated with Trauma Severity

The patterns of cell viability and death in CD66b^+^ granulocytes, total T lymphocytes, and CD4^+^ and CD8^+^ T-cell subsets are presented in Table 2. The proportion of early apoptotic CD66b^+^ granulocytes was significantly lower in the severe trauma group than in the non-severe trauma group (2.9% [1.4–6.7] vs. 6.3% [3.7–10.9], *p* = 0.001). Although the proportion of live CD66b^+^ granulocytes was numerically higher in the severe trauma group, this difference was not statistically significant (91.2% [86.3–93.9] vs. 88.3% [82.2–91.8], *p* = 0.063). The rates of late apoptosis and necrosis of CD66b^+^ granulocytes did not differ significantly between the groups.

By contrast, no significant between-group differences were observed in total T lymphocytes or CD8^+^ T-cell subsets. Among CD4^+^ T lymphocytes, the proportion of necrotic cells was higher in the severe trauma group than in the non-severe trauma group (4.9% [3.6–6.5] vs. 3.5% [2.6–4.5], *p* = 0.017), whereas the proportions of live, early apoptotic, and late apoptotic cells did not differ significantly between the groups.

### 3.3. Comparison of Circulating Inflammatory Mediator Levels Between Severe and Non-Severe Trauma

**Figure 3:**
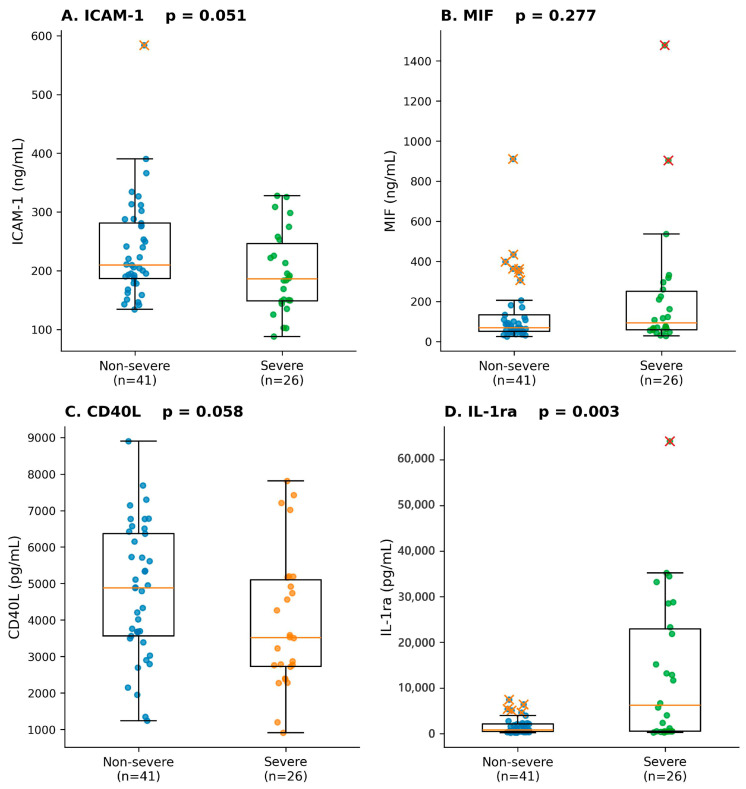
Serum ICAM-1, MIF, CD40L, and IL-1ra concentrations according to trauma severity. The original measured values are presented on linear y-axes. Box plots show medians and interquartile ranges. Crosses indicate outliers. Between-group comparisons were performed using the Mann–Whitney U test. ICAM-1, intercellular adhesion molecule-1; MIF, macrophage migration inhibitory factor; CD40L, CD40 ligand; IL-1ra, interleukin-1 receptor antagonist.

As shown in Figure 3, the IL-1ra levels were significantly higher in the severe trauma group than in the non-severe trauma group (6199.90 [511.83–22,948.33] pg/mL vs. 768.45 [444.93–2087.69] pg/mL, *p* = 0.003). The ICAM-1 and CD40L levels were lower in the severe trauma group, although the differences were not significant (*p* = 0.051 and *p* = 0.058, respectively). MIF levels did not differ significantly between the groups (*p* = 0.277).

### 3.4. Relationship Between ISS and Circulating Inflammatory Mediator Levels

Multiple linear regression analysis was performed to evaluate the association between ISS and circulating levels of inflammatory mediators (Table 3). In the mediator-only model, log-transformed IL-1ra levels were significantly associated with ISS (β = 3.00, 95% CI 1.56–4.44, *p* < 0.001). This association remained significant in the adjusted sensitivity model including age, sex, injury-to-sampling time, GCS, and systolic blood pressure (β = 2.26, 95% CI 0.65–3.87, *p* = 0.007). No significant associations were observed for log-transformed ICAM-1, MIF, or CD40L in the adjusted sensitivity model.

## 4. Discussion

In this study, severe trauma was associated with a lower proportion of early apoptotic CD66b^+^ granulocytes and higher circulating IL-1ra levels immediately after injury. The proportion of live CD66b^+^ granulocytes was numerically higher in the severe trauma group, although this difference was not statistically significant. The rates of late apoptosis and necrosis of CD66b^+^ granulocytes were similar between the groups. In the adaptive immune compartment, most T-cell death parameters did not differ significantly according to trauma severity, although the proportion of necrotic CD4^+^ T lymphocytes increased in the severe trauma group. Among the inflammatory mediator levels measured, only IL-1ra was significantly associated with ISS.

The main cell death-related finding of this study was the selective reduction in early apoptotic CD66b^+^ granulocytes in patients with severe trauma. The live, late apoptotic, and necrotic CD66b^+^ granulocyte fractions did not differ significantly between the groups; this may indicate a selective difference in the early apoptotic status of the CD66b^+^ granulocyte compartment rather than a generalized alteration in granulocyte death. This selective pattern may be biologically plausible in the context of rapid innate immune activation after tissue injury [7]. Consistent with this interpretation, previous studies have reported alterations in granulocyte or polymorphonuclear leukocyte apoptosis after injury [12,24], and neutrophil-focused studies have shown that major trauma can affect neutrophil phenotype, function, and apoptotic responses shortly after injury [11,13,14]. These observations provide a biological context for the present finding, but this interpretation should remain confined to the CD66b^+^ granulocyte compartment analyzed in this study. The flow cytometry panel identified this population as CD45^+^CD66b^+^CD3^−^ cells and did not distinguish neutrophils, eosinophils, or granulocyte maturation states; therefore, the reduced early apoptotic fraction should not be interpreted as direct evidence of a neutrophil-specific mechanism. Given that post-traumatic immune responses and leukocyte cell-death regulation may change over time [4,14], this finding should be interpreted within the hyperacute sampling window rather than as evidence of the subsequent cell-death trajectory after injury.

Notably, trauma severity was not associated with broad differences in cell-death patterns among total T lymphocytes or CD8^+^ T-cell subsets. This may partly reflect the temporal characteristics of post-traumatic immune responses: innate immune activation occurs rapidly after tissue injury, whereas adaptive immune alterations develop over time, manifesting as changes in lymphocyte count, subset composition, and functional responsiveness [7,15,16]. Therefore, adaptive immune dysfunction after trauma may not be fully captured by viability or apoptosis measurements alone [15,16]. Nevertheless, the proportion of necrotic CD4^+^ T lymphocytes was higher in the severe trauma group. Because no significant differences were observed in other CD4^+^ T-cell death states and no adjustment for multiple comparisons was applied, this finding should be interpreted cautiously as an exploratory, subset-specific signal of CD4^+^ T-cell injury after severe trauma. Severe trauma can alter CD4^+^ T-cell proliferation and regulatory T-cell activity, and recent evidence indicates that non-apoptotic cell-death pathways, such as ferroptosis, may contribute to CD4^+^ T-cell depletion after severe polytrauma [25,26]. Although these findings provide biological context, the increased CD4^+^ T-cell necrotic fraction observed in this study requires further functional and mechanistic validation.

Interestingly, IL-1ra levels were significantly elevated in the severe trauma group and remained significantly associated with ISS in both the mediator-only model and the adjusted sensitivity model that included age, sex, injury-to-sampling time, GCS, and systolic blood pressure. IL-1ra is an endogenous anti-inflammatory mediator that inhibits IL-1 signaling by competitively binding to IL-1 receptors [27]. IL-1ra levels increase early after injury, and higher early IL-1ra levels are associated with injury burden, organ dysfunction, and mortality [8,17,28,29]. In this context, the observed elevation of IL-1ra may reflect early activation of counter-regulatory anti-inflammatory responses accompanying the initial systemic inflammatory reaction after severe injury [4,7,8]. This interpretation is supported by the findings of studies linking early IL-1ra elevation and inflammatory mediator profiles to subsequent organ dysfunction after major trauma [28,30]. However, although the association between IL-1ra and ISS suggests that IL-1ra levels may reflect the magnitude of early injury-related immune activation, the broad interquartile range observed in the severe trauma group indicates substantial inter-individual variability. Therefore, these findings suggest that IL-1ra may be a severity-associated marker of early injury-related immune activation in this cohort, but they do not establish IL-1ra as an independent predictor, causal mediator, or clinically validated biomarker of subsequent outcomes.

In contrast to IL-1ra, ICAM-1, CD40L, and MIF did not differ significantly according to trauma severity. Although ICAM-1 and CD40L levels were lower in the severe trauma group, these differences were not significant. Recent trauma biomarker studies have suggested that the early prognostic relevance of endotheliopathy-related markers may depend on specific markers, sampling timing, and clinical context [31]. Therefore, the absence of a significant ICAM-1 difference in the present study may reflect variability in early endothelial activation markers after trauma as well as the hyperacute timing of blood sampling. CD40L levels are linked to platelet activation and thromboinflammatory responses, and prior trauma data have associated high early sCD40L levels with tissue injury, shock, coagulopathy, and mortality [18]. However, the absence of a significant difference in CD40L levels in the present study should be interpreted cautiously, because circulating CD40L levels can be influenced by sampling timing, platelet activation status, and platelet-related thromboinflammatory dynamics. As platelet activation and serial mediator changes were not directly assessed, the present findings do not support conclusions regarding the role of CD40L in the hyperacute response to trauma. Furthermore, MIF levels did not differ significantly between the groups. Although previous studies found associations between MIF and trauma-related inflammation, infectious complications, and organ dysfunction [19,32,33], its significance in the immediate post-injury phase may vary according to injury pattern, sampling timing, and downstream immune responses.

This study had some limitations. First, this was a single-center study with a relatively small sample size. The study size was determined by feasibility rather than by a priori power calculation; therefore, the study may have been underpowered to detect modest differences in T-cell subsets or selected inflammatory mediators and to support fully adjusted multivariable models. In addition, because a prospective screening log was not maintained, the total number of screened patients, potentially eligible non-enrolled patients, and reasons for non-participation could not be fully reconstructed. This may have introduced selection bias toward patients in whom consent and research blood sampling within 1 h of emergency department arrival were feasible. Therefore, these findings should be interpreted as representing the enrolled analytic cohort rather than the entire emergency department trauma population. Second, all measurements were obtained within 1 h of arrival at the emergency department; therefore, changes in immune responses over time could not be assessed. Third, although cell viability and death patterns were assessed, functional immune activity and relevant signaling pathways were not directly evaluated. Fourth, in the flow cytometric analysis, the CD66b^+^ granulocyte compartment was evaluated rather than discrete granulocyte subsets; therefore, the present study did not distinguish neutrophils from other CD66b-expressing granulocyte populations or define maturation-state-specific death patterns. Additionally, despite the use of a consistent gating strategy, the classification of cell death states may have been influenced by sampling timing, staining conditions, or gating thresholds. Endothelial and platelet functions were not directly assessed, which limits the interpretation of the observed ICAM-1 and CD40L findings. Finally, residual confounding due to injury mechanisms, associated injuries, transfusion, operative intervention, comorbidities, and other clinical factors could not be excluded.

To address these limitations, future multicenter studies with larger sample sizes, serial immune measurements at 0–1 h, 6 h, 24 h, and 72 h, and longitudinal clinical follow-up are needed to determine whether these hyperacute immune alterations persist over time and are associated with major clinical outcomes, including infection, multiple organ dysfunction, and mortality. Additional mechanistic studies incorporating granulocyte or neutrophil subpopulation analysis, maturation-state profiling, neutrophil functional assays, inflammatory signaling assessment, and single-cell transcriptomic approaches may further clarify the biological mechanisms underlying these early immune responses.

## 5. Conclusions

In this study, severe trauma was associated with a lower proportion of early apoptotic CD66b^+^ granulocytes and higher circulating IL-1ra levels shortly after injury. Broad differences in T-cell death patterns were not observed, although an exploratory increase in CD4^+^ T-cell necrosis was noted in the severe trauma group. Among the measured inflammatory mediators, IL-1ra was significantly associated with ISS in the regression analyses, suggesting that it may reflect the magnitude of early injury-related immune activation in this cohort. These findings indicate that early immune alterations after severe trauma may involve compartment-specific changes in CD66b^+^ granulocyte death patterns and counter-regulatory inflammatory responses, rather than generalized changes across all leukocyte populations.

## Figures and Tables

**Table 1:** Baseline characteristics of patients in each trauma severity group.

Variable	Non-Severe Group (*n* = 41)	Severe Group (*n* = 26)	*p*-Value
Male sex, n (%)	30 (73.2%)	19 (73.1%)	1.000
Age, years	54.8 ± 18.2	54.8 ± 19.8	0.998
GCS	15.0 [15.0–15.0]	15.0 [10.5–15.0]	0.001
ISS	5.0 [2.0–10.0]	22.0 [20.3–28.5]	<0.001
Systolic blood pressure, mmHg	140.0 [126.0–145.0]	137.0 [110.0–155.0]	0.608
Diastolic blood pressure, mmHg	83.9 ± 14.0	81.0 ± 16.0	0.445
Heart rate, bpm	90.6 ± 14.6	82.5 ± 19.9	0.065
Respiratory rate, breaths/min	20.0 [20.0–20.0]	20.0 [20.0–23.5]	0.031
Body temperature, °C	36.7 ± 0.6	36.4 ± 0.5	0.053
Time from injury occurrence to ED arrival, min	41.0 [33.0–53.0]	41.5 [30.2–53.0]	0.541
Time from ED arrival to blood sampling, min	45.0 ± 7.9	47.5 ± 5.1	0.144
Time from injury occurrence to blood sampling, min	85.0 [76.0–105.0]	86.5 [75.5–105.2]	0.949
Hospital admission, n (%)	27 (65.9%)	25 (96.2%)	0.009
ICU admission, n (%)	12 (29.3%)	23 (88.5%)	<0.001
Operation, n (%)	19 (46.3%)	15 (57.7%)	0.513

Data are presented as means ± standard deviations, medians [interquartile ranges], or n (%), as appropriate. *p*-values were calculated using the chi-square test or Fisher’s exact test for categorical variables and the independent *t*-test or Mann–Whitney U test for continuous variables, as appropriate. GCS, Glasgow Coma Scale; ISS, Injury Severity Score; ED, emergency department; ICU, intensive care unit.

**Table 2:** Comparison of cell viability and cell death between patients with non-severe and severe trauma.

Cell Type	Cell State	Non-Severe Trauma Group (*n* = 41), %	Severe Trauma Group (*n* = 26), %	*p*-Value
CD66b^+^ granulocyte	Live	88.3 [82.2–91.8]	91.2 [86.3–93.9]	0.063
	Early apoptosis	6.3 [3.7–10.9]	2.9 [1.4–6.7]	0.001
	Late apoptosis	0.7 [0.5–1.2]	0.6 [0.5–1.4]	0.684
	Necrosis	3.5 [2.4–5.2]	4.4 [2.6–5.4]	0.364
T lymphocyte	Live	87.4 [80.5–92.7]	84.8 [79.4–91.7]	0.567
	Early apoptosis	6.3 [2.7–11.5]	7.1 [1.9–11.7]	0.944
	Late apoptosis	1.9 [0.6–4.7]	2.2 [0.7–3.9]	1.000
	Necrosis	3.7 [2.6–4.0]	4.2 [2.8–5.8]	0.050
CD4^+^ T lymphocyte	Live	85.8 [80.3–93.0]	82.2 [76.3–88.3]	0.290
	Early apoptosis	6.6 [1.7–10.4]	9.7 [2.5–12.5]	0.454
	Late apoptosis	2.2 [0.4–5.5]	2.5 [1.5–5.1]	0.736
	Necrosis	3.5 [2.6–4.5]	4.9 [3.6–6.5]	0.017
CD8^+^ T lymphocyte	Live	88.7 [80.0–92.0]	87.4 [78.9–90.9]	0.726
	Early apoptosis	6.2 [3.2–14.3]	6.9 [3.6–13.3]	0.829
	Late apoptosis	1.6 [0.4–2.7]	1.4 [0.6–4.0]	0.871
	Necrosis	3.4 [2.3–4.3]	3.3 [2.1–5.0]	0.930

Data are presented as medians [interquartile ranges]. *p*-values were calculated using the Mann–Whitney U test and were not adjusted for multiple comparisons.

**Table 3:** Multiple linear regression analysis of circulating inflammatory mediator levels and ISS in mediator-only and adjusted sensitivity models.

Variable	Mediator-Only Model β Coefficient (95% CI)	*p*-Value	Adjusted Sensitivity Model β Coefficient (95% CI)	*p*-Value
log(ICAM-1)	−5.24 (−11.64 to 1.17)	0.107	−5.01 (−11.54 to 1.52)	0.130
log(MIF)	1.20 (−1.22 to 3.61)	0.326	−0.12 (−2.76 to 2.52)	0.928
log(CD40L)	−3.64 (−8.09 to 0.81)	0.107	−4.61 (−9.28 to 0.05)	0.053
log(IL-1ra)	3.00 (1.56 to 4.44)	<0.001	2.26 (0.65 to 3.87)	0.007

The mediator-only model included log-transformed ICAM-1, MIF, CD40L, and IL-1ra levels. The adjusted sensitivity model additionally included age, sex, injury-to-sampling time, GCS, and systolic blood pressure. ISS, Injury Severity Score; GCS, Glasgow Coma Scale; ICAM-1, intercellular adhesion molecule-1; MIF, macrophage migration inhibitory factor; CD40L, CD40 ligand; IL-1ra, interleukin-1 receptor antagonist; CI, confidence interval.

## Data Availability

The data presented in this paper are available upon reasonable request from the corresponding author. The data are not publicly available due to privacy and ethical restrictions related to patient-level clinical and laboratory data.

## Supplementary Materials

The following supporting information can be downloaded at: https://www.mdpi.com/article/10.3390/jcm15114371/s1, Table S1. STROBE Checklist.
