# Supplementary material for: Early Immune Alterations in Adult Patients with Trauma According to Injury Severity: Cell-Death Patterns and Inflammatory Mediator Profiles
Source: J Clin Med. 2026 Jun 5;15(11):4371. doi: 10.3390/jcm15114371 (PMC13258606; doi:10.3390/jcm15114371)
Supplement: Supplementary file 1 [file jcm-15-04371-s001.zip › jcm-4331647-supplementary.pdf]

Supplementary Table S1. STROBE Checklist

Manuscript title: Early Immune Alterations in Adult Patients with Trauma According to Injury Severity: Cell-Death Patterns and Inflammatory Mediator Profiles

Section/topic	Item No.	Recommendation	Page No.	Relevant text from manuscript
Title and abstract	1(a)	Indicate the study's design with a commonly used term in the title or the abstract	p. 2 (Abstract, Methods)	"This single-center prospective observational study included 67 adult patients with trauma..."
Title and abstract	1(b)	Provide in the abstract an informative and balanced summary of what was done and what was found	pp. 2–3 (Abstract)	The abstract summarizes the background/objectives, methods, results, and conclusions, including the comparison of cell viability and death patterns, mediator measurements, and the main findings.
Introduction	2	Explain the scientific background and rationale for the investigation being reported	pp. 4–5 (Introduction)	The Introduction describes the clinical relevance of early immune responses after trauma, the roles of granulocytes and T-cell subsets, and the rationale for selected circulating mediators.
Introduction	3	State specific objectives, including any prespecified hypotheses	p. 5 (Introduction, final paragraph)	"The primary objective was to compare cell viability and cell-death patterns between patients with non-severe trauma (ISS < 15) and those with severe trauma (ISS ≥ 15). The secondary objectives were..."
Methods	4	Present key elements of study design early in the paper	p. 6 (Methods, Study Design and Setting)	"This was a single-center, prospective, observational study conducted in the emergency department of Korea University Guro Hospital..."
Methods	5	Describe the setting, locations, and relevant dates, including periods of recruitment, exposure, follow-up, and data collection	p. 6 (Methods, Study Design and Setting)	The setting, hospital characteristics, recruitment period (January 2023 to September 2025), and emergency department context are described.
Methods	6(a)	Give the eligibility criteria, and the sources and methods of selection of participants	pp. 6–7 (Methods, Study Design and Setting)	Adult patients aged ≥19 years were eligible if they met prespecified red criteria or yellow mechanism-of-injury criteria. Patients were enrolled when consent and blood sampling within 1 h of ED arrival were completed; otherwise they were not enrolled.
Methods	6(b)	For matched studies, give matching criteria and number of exposed and unexposed	N/A	Not applicable. This was not a matched study.
Methods	7	Clearly define all outcomes, exposures, predictors, potential confounders, and effect modifiers. Give diagnostic criteria, if applicable	pp. 8 and 11 (Laboratory Variables; Statistical Analysis)	Laboratory variables, ISS-based trauma severity groups, circulating mediators, and adjusted model covariates (age, sex, injury-to-sampling time, GCS, and systolic blood pressure) are defined.
Methods	8	For each variable of interest, give sources of data and details of methods of assessment (measurement)	pp. 6–11 (Methods)	Clinical data collection, blood sampling, flow cytometric staining and gating, Annexin V/PI-defined cell-death states, ELISA mediator measurements, and statistical methods are described.
Methods	9	Describe any efforts to address potential sources of bias	pp. 6–11 and p. 21 (Methods; Limitations)	The manuscript describes standardized sampling and acquisition protocols, laboratory blinding to trauma severity and clinical outcomes, consistent gating templates, and acknowledges possible selection bias due to absence of a prospective screening log.
Methods	10	Explain how the study size was arrived at	p. 11 (Study Size)	"Directly applicable preliminary data were not available... therefore a formal a priori sample size calculation was not performed. The study size was thus determined by feasibility..."
Methods	11	Explain how quantitative variables were handled in the analyses. If applicable, describe which groupings were chosen and why	pp. 8 and 11 (Group Classification; Statistical Analysis)	Continuous variables were assessed for normality using the Shapiro–Wilk test and summarized as means ± standard deviations or medians [interquartile ranges], as appropriate. Trauma severity was categorized using an ISS cutoff of 15 to define non-severe and severe trauma groups. Skewed inflammatory mediator concentrations were log-transformed before regression analysis
Methods	12(a)	Describe all statistical methods, including those used to control for confounding	p. 11 (Statistical Analysis)	The manuscript describes chi-square/Fisher's exact tests, independent t-test/Mann–Whitney U

				test, Shapiro–Wilk normality testing, log transformation, multiple linear regression, and adjusted sensitivity analysis.
Methods	12(b)	Describe any methods used to examine subgroups and interactions	p. 11 (Statistical Analysis)	“No subgroup or interaction analyses were performed.”
Methods	12(c)	Explain how missing data were addressed	p. 11 (Statistical Analysis)	“There were no missing data for the variables included in the final analyses; therefore, no imputation was performed.”
Methods	12(d)	If applicable, explain how loss to follow-up was addressed / matching / sampling strategy	N/A	Not applicable. This laboratory-based observational study did not involve follow-up analyses or matched groups.
Methods	12(e)	Describe any sensitivity analyses	p. 11 (Statistical Analysis)	“For sensitivity analysis, an adjusted model was fitted by including age, sex, injury-to-sampling time, GCS, and systolic blood pressure.”
Results	13(a)	Report numbers of individuals at each stage of study	pp. 7 and 12 (Figure 1; Results)	Figure 1 summarizes the enrolled analytic cohort. Results state that 67 patients were included and classified into non-severe (n = 41) and severe (n = 26) groups.
Results	13(b)	Give reasons for non-participation at each stage	pp. 6–7 and 21 (Methods; Limitations)	The Methods states that patients for whom consent and research sampling could not be completed within the 1-h window were not enrolled. It also states that a prospective screening log was not maintained; the limitation is acknowledged.
Results	13(c)	Consider use of a flow diagram	p. 7 (Figure 1)	Figure 1 presents the flow diagram of the enrolled analytic cohort.
Results	14(a)	Give characteristics of study participants and information on exposures and potential confounders	pp. 12–14 (Table 1)	Table 1 presents age, sex, GCS, ISS, vital signs, injury-to-arrival and sampling intervals, admission, ICU admission, and operation according to trauma severity.
Results	14(b)	Indicate number of participants with missing data for each variable of interest	p. 11 (Statistical Analysis)	The manuscript states that there were no missing data for variables included in the final analyses.
Results	14(c)	Cohort study—Summarise follow-up time	N/A	Not applicable. The study did not analyze longitudinal follow-up outcomes.
Results	15	Report numbers of outcome events or summary measures over time	pp. 12–17 (Tables 1–3; Figure 3)	Baseline characteristics, cell viability and death patterns, mediator concentrations, and regression results are reported. Longitudinal outcomes over time were not assessed.
Results	16(a)	Give unadjusted estimates and, if applicable, confounder-adjusted estimates and their precision	pp. 15–17 (Table 2, Figure 3, Table 3)	Between-group comparisons and p-values are provided. Table 3 provides mediator-only and adjusted sensitivity regression coefficients with 95% CIs and p-values.
Results	16(b)	Report category boundaries when continuous variables were categorized	pp. 8 and 12 (Group Classification; Results)	ISS < 15 defined the non-severe trauma group and ISS ≥ 15 defined the severe trauma group.
Results	16(c)	If relevant, consider translating estimates of relative risk into absolute risk	N/A	Not applicable. The study did not estimate relative risks for clinical outcomes.
Results	17	Report other analyses done	pp. 16–17 (Relationship Between ISS and Circulating Inflammatory Mediator Levels; Table 3)	The mediator-only and adjusted sensitivity regression analyses are reported. No subgroup or interaction analyses were performed.
Discussion	18	Summarise key results with reference to study objectives	p. 18 (Discussion, first paragraph)	The Discussion summarizes reduced early apoptotic $CD66b^{+}$ granulocytes, elevated IL-1ra, exploratory $CD4^{+}$ T-cell necrosis, and the mediator-ISS association.
Discussion	19	Discuss limitations, taking into account sources of potential bias or imprecision	pp. 21–22 (Limitations paragraph)	The manuscript discusses small single-center sample, feasibility-based study size, lack of full screening denominator, selection bias, single time-point measurement, lack of functional assays, $CD66b^{+}$ compartment limitations, and residual confounding.
Discussion	20	Give a cautious overall interpretation considering objectives, limitations, multiplicity, similar studies, and other evidence	pp. 18–22 (Discussion and Conclusions)	The Discussion distinguishes main findings from exploratory observations, notes unadjusted multiple comparisons, limits interpretation of IL-1ra, and confines $CD66b^{+}$ findings to the granulocyte compartment.
Discussion	21	Discuss the generalisability (external	p. 21 (Limitations	The manuscript states that findings should be

		validity) of the study results	paragraph)	interpreted as representing the enrolled analytic cohort rather than the entire emergency department trauma population.
Other information	22	Give the source of funding and the role of the funders	p. 23 (Funding)	The Funding section states that the research and APC were funded by Korea University and that the funder had no role in study design, data collection, analysis, interpretation, manuscript preparation, or publication decision.
